# Hsp27 participates in the maintenance of breast cancer stem cells through regulation of epithelial-mesenchymal transition and nuclear factor-κB

**DOI:** 10.1186/bcr3042

**Published:** 2011-10-24

**Authors:** Li Wei, Tsung-Ta Liu, Hsiu-Huan Wang, Hui-Mei Hong, Alice L Yu, Hsiang-Pu Feng, Wen-Wei Chang

**Affiliations:** 1 Department of Neurosurgery, Taipei Medical University-WanFang Hospital, Hsing-Long Rd, Sec. 3, No.111, Taipei City, 116, Taiwan; 2 Department of Neurosurgery, Taipei Medical University-Shuang Ho Hospital, Jhongjheng Rd., No.291, New Taipei City, 23561, Taiwan; 3 Department of Nephrology, Taipei Medical University-Shuang Ho Hospital, Jhongjheng Rd., No.291, New Taipei City, 23561, Taiwan; 4 School of Biomedical Sciences and Department of Medical Research, Chung Shan Medical University, Chien-Kuo N. Rd., Sec. 1, No.110, Taichung City, 402, Taiwan; 5 The Genomics Research Center, Academia Sinica, Academia Rd., Sec. 2, No. 128, Taipei City, 115, Taiwan

## Abstract

**Introduction:**

Heat shock proteins (HSPs) are normally induced under environmental stress to serve as chaperones for maintenance of correct protein folding but they are often overexpressed in many cancers, including breast cancer. The expression of Hsp27, an ATP-independent small HSP, is associated with cell migration and drug resistance of breast cancer cells. Breast cancer stem cells (BCSCs) have been identified as a subpopulation of breast cancer cells with markers of CD24-CD44+ or high intracellular aldehyde dehydrogenase activity (ALDH+) and proved to be associated with radiation resistance and metastasis. However, the involvement of Hsp27 in the maintenance of BCSC is largely unknown.

**Methods:**

Mitogen-activated protein kinase antibody array and Western blot were used to discover the expression of Hsp27 and its phosphorylation in ALDH + BCSCs. To study the involvement of Hsp27 in BCSC biology, siRNA mediated gene silencing and quercetin treatment were used to inhibit Hsp27 expression and the characters of BCSCs, which include ALDH+ population, mammosphere formation and cell migration, were analyzed simultaneously. The tumorigenicity of breast cancer cells after knockdown of Hsp27 was analyzed by xenograftment assay in NOD/SCID mice. The epithelial-mesenchymal transition (EMT) of breast cancer cells was analyzed by wound-healing assay and Western blot of snail, vimentin and E-cadherin expression. The activation of nuclear factor kappa B (NF-κB) was analyzed by luciferase-based reporter assay and nuclear translocation.

**Results:**

Hsp27 and its phosphorylation were increased in ALDH+ BCSCs in comparison with ALDH- non-BCSCs. Knockdown of Hsp27 in breast cancer cells decreased characters of BCSCs, such as ALDH+ population, mammosphere formation and cell migration. In addition, the *in vivo *CSC frequency could be diminished in Hsp27 knockdown breast cancer cells. The inhibitory effects could also be observed in cells treated with quercetin, a plant flavonoid inhibitor of Hsp27, and it could be reversed by overexpression of Hsp27. Knockdown of Hsp27 also suppressed EMT signatures, such as decreasing the expression of snail and vimentin and increasing the expression of E-cadherin. Furthermore, knockdown of Hsp27 decreased the nuclear translocation as well as the activity of NF-κB in ALDH + BCSCs, which resulted from increasing expression of IκBα. Restored activation of NF-κB by knockdown of IκBα could reverse the inhibitory effect of Hsp27 siRNA in suppression of ALDH+ cells.

**Conclusions:**

Our data suggest that Hsp27 regulates the EMT process and NF-κB activity to contribute the maintenance of BCSCs. Targeting Hsp27 may be considered as a novel strategy in breast cancer therapy.

## Introduction

Heat shock proteins (HSPs) are a group of proteins that were first discovered under heat shock or other chemical stimulus in a wide range of species and function as molecular chaperones that can interact with their substrates to shift the balance from denatured protein conformation toward functional conformation [1]. Besides their chaperone function, HSPs have been reported to be overexpressed in various cancers and to display a correlation with patients' survival or response to therapy in specific cancer types and may serve as novel therapeutic targets [2]. Hsp27 belongs to a small HSP family and has been found to contribute to the malignant properties of cancer cells, including increased tumorigenicity, treatment resistance and apoptosis inhibition [3]. In breast cancer, Hsp27 has been reported as a risk factor of malignant progression in benign proliferating breast lesions [4] and its expression could help to differentiate benign and malignant breast lesions in fine needle aspirate [5]. Hsp27 has been reported to be associated with drug resistance and cell mobility properties of breast cancer. In the Herceptin-resistant SKBR3 breast cancer cell line, silencing of Hsp27 expression by siRNA increased the susceptibility to Herceptin treatment through decreasing Her2 protein stability [6]. Overexpression of Hsp27 also protected MDA-MB-231 breast cancer cells from doxorubicin induced apoptosis [7]. Inhibition of Hsp27 phosphorylation with a small molecule inhibitor also suppressed the cell invasion capacity of metastatic MDA-MB-231 cells [8]. Although Hsp27 is involved in chemoresistance and invasion phenotypes of breast cancer cell lines, the involvement of Hsp27 in breast cancer stem cells (BCSCs) is not fully understood.

Cancer stem cells, which are a particular subset of cancer cells responsible for tumorigenesis, chemoresistance and metastasis, are emerging targets in cancer research [9]. In breast cancer, BCSCs have been identified as cells with surface markers of CD24-CD44+ [10] or high intracellular aldehyde dehyprogenase activity (ALDH+) [11]. Recently, Hsp27 has been proven to contribute to the drug resistance property of lung cancer stem cells [12]. The expression of Hsp27 was increased in lung CSCs treated with cisplatin/gemcitabine. A combination of chemotherapy with a plant flavonoid compound quercetin, which can inhibit Hsp27 expression, could suppress the tumor growth as well as the expression of stemness genes, including *Oct4*, *Nanog *and *Sox2 *[12]. Quercetin could also sensitize epigallocathechin gallate to inhibit the spheroid formation, cell survival and invasion of CD44+ CD133+ prostate cancer stem cells [13], although the detailed molecular mechanisms remains unknown.

In the present study, we identified that the expression of Hsp27 and its phosphorylation were increased in ALDH+ BCSCs. Inhibition of Hsp27 by siRNA or quercetin, a plant flavonoid compound, suppressed characters of BCSCs, including ALDH+ population, mammosphere formation and epithelial-mesenchymal transition (EMT). We also found that Hsp27 could regulate the NF-kB activity of BCSCs. These findings suggest that Hsp27 regulates the maintenance of BCSCs and it may serve as a potential target in future breast cancer therapy.

## Materials and methods

### Cell culture and reagents

AS-B145 and AS-B244 breast cancer cells, which derived from BC0145 or BC0244 xenograft human breast cancer cells [14], were cultured in MEMα supplemented with 10% fetal bovine serum, bovine insulin (0.1 mg/mL), sodium pyruvate (1 mM), and Glutamax (2 mM). 4T1 mouse breast cancer cells and an MDA-MB-231 human breast cancer cell line were obtained from ATCC (Manassas, VA, USA) and cultivated as ATCC's recommendation. The cells were maintained in a 5% CO_2 _air humidified atmosphere at 37°C. Quercetin and JSH-23 were purchased from Calbiochem (San Diego, CA, USA) and dissolved in dimethyl sulfoxide (DMSO). pDsRed-Express2-C1 vector was purchased from Clontech (Mountain View, CA, USA). To construct DsRed tagged Hsp27, the *Hsp27 *gene was cloned from AS-B145 cDNA by the following primers: 5'-CACTTGAGATCTACCGAGCGCCGCGTCCCCTTC-3' (forward); 5'-CAAGTGGAATTCTTACTTGGCGGCAGTCTCATC-3' (reverse) and inserted into pDsRed-Express2-C1 vector by BglII and EcoRI restriction sites (pDsRed-Hsp27).

### Antibody array and Western blot

MAPK antibody array was purchased from R&D Systems (Minneapolis, MN, USA) and conducted following the manufacturer's protocol. Briefly, the membrane was blocked in blocking buffer and incubated with 150 μg of total cellular protein and detection antibody simultaneously at 4°C overnight. After washing, the membrane was further incubated with streptavidin-HRP at room temperature for 30 minutes and a signal was developed with ECL substrate. For Western blot, cells were lysed with NP-40 lysis buffer and 25 μg of total protein were separated by SDS-PAGE and transferred to polyvinylidene fluoride membrane. Protein detection was conducted by SignalBoost™ Immunodetection Enhancer kit (Calbiochem) according to the manufacturer's recommendation. Hsp27 antibody was purchased from Stressgen (Ann Arbor, MI, USA). IκBα and phosphor-IκBα antibodies were purchased from Cell Signaling Technologies (Boston, MA, USA). NF-κB p65 antibody was purchased from Millipore (Billerca, MA, USA). Snail, twist, vimentin, GAPDH and histone H1 antibodies were purchased from Santa Cruz Biotechnology (Santa Cruz, CA, USA). β-actin antibody was purchased from Novus Biologicals (Littleton, CO, USA).

### RNA interference and Hsp27 overexpression

The specific siRNA oligos of *Hsp27 *(sc-29350) or *IκBα *(sc-29360), or negative control siRNA oligos was purchased from Santa Cruz Biotechnologies, Inc. The siRNA oligos of *Hsp27 *or *IκBα *consisted of pools of three target-specific siRNAs designed to knockdown gene expression and the target sequences were listed below:

sc-29350A: Sense: GAGUGGUCGCAGUGGUUAGtt; Antisense: CUAACCACUGCGACCACUCttsc-29350B: Sense: GACGAGCUGACGGUCAAGAtt; Antisense: UCUUGACCGUCAGCUCGUCttsc-29350C: Sense: CCACGCAGUCCAACGAGAUtt; Antisense: AUCUCGUUGGACUGCGUGGtt

sc-29360A: Sense: CCACACGUGUCUACACUUAtt; Antisense: UAAGUGUAGACACGUGUGGttsc-29360B: Sense: GACGAGAAAGAUCAUUGAAtt; Antisense: UUCAAUGAUCUUUCUCGUCttsc-29360C: Sense: CCUGUCAAGGUUUGUGUUAtt; Antisense: UAACACAAACCUUGACAGGttMetafecteneSI transfection reagent (Biontex, Martinsired, Germany) was used for siRNA transfection following the manufacturer's protocol. To overexpress Hsp27, cells were transfected with pDsRed-Hsp27 by MetafectenePro transfection reagent (Biontex) as a ratio (DNA (μg):reagent (μl)) of 1:3.

### ALDEFLUOR assay

An ALDEFLUOR assay kit was purchased from StemCell Technologies, Inc. (Vancouver, BC, Canada) and used following the manufacturer's recommendations. Briefly, 1 × 10^5 ^cells were suspended in 50 μl of assay buffer and added to BODIPY-aminoacetaldehyde substrate to a final concentration of 1 μM. For ALDH1 inhibitor control, diethylaminobenzaldehyde (DEAB) was added to the final concentration of 150 μM. Cells were then incubated at 37°C for 45 minutes and stained with 7-AAD on ice for a further 5 minutes. After being washed with PBS, green fluorescence positive cells in live cells (7AAD-) were analyzed by flow cytometry (Epics XL, Beckman Coulter, Brea, CA, USA) by comparing the fluorescence intensity of the DEAB treated sample; these cells will be represented as cells with high ALDH activity (ALDH+ cells).

### Mammosphere culture

Cells were harvested from monolayer culture or collected by fluorescence-activated cell sorting (FACS) and prepared at a density of 1 × 10^4 ^cells/ml in DMEM/F12 medium contain 0.5% methylcellulose, 0.4% bovine serum albumin, 10 ng/ml EGF, 10 ng/ml bFGF, 5 μg/ml insulin, 1 μM hydrocortisone and 4 μg/ml heparin. A total of 2 ml of cell solution was seeded into wells of ultralow attachment six-well-plate (Corning, Lowell, MA, USA) and incubated for seven days. For secondary spheres, the cells were collected from accutase treated primary spheres, seeded at a density of 2,500 cells/ml and cultivated for a further seven days.

### Xenograftment assay in NOD/SCID mice

The tumorigenicity of AS-B145 sphere cells was examined by xenograftment assay in NOD/SCID mice. After transfection with ctrl-siRNA or si-Hsp27 for 48 h, an indicated number of AS-B145 cells was mixed with 10^5 ^normal breast fibroblasts by 50 μl of MEMα:matrigel (1:1) (BD Biosciences, San Jose, CA, USA) and injected into mammary fat pads of female NOD/SCID mice (BioLASCO Taiwan Co., Ltd. Taipei, Taiwan). The tumor formation was monitored weekly. The CSC frequency was calculated by Extreme Limiting Dilution Assay (Walter-Eliza Hall Bioinformatics, Bundoora Victoria, Australia.) [15].

### Cell migration assay

A cell migration assay was conducted by Oris Universal Cell Migration Assembly kit (Platypus Technologies, LCC, Madison, WI, USA) following the manufacturer's protocol. Briefly, 5 × 10^4 ^cells/well/100 μl were loaded into stopper-loaded wells and incubated overnight to permit cell attachment. To start cell migration, the stoppers were removed; wells were gently washed with PBS, then added to complete cell culture medium and incubated for 16 to 18 h. Pictures of wells were captured with inverted microscopy after fixation and stain with 0.5% crystal violet/50% EtOH. Data were analyzed with ImageJ software (National Health Institute, Bethesda, MD, USA).

### NF-kB reporter assay

The luciferase based NF-κB reporter vector was obtained from Stratagene (Santa Clara, CA, USA). The assay was conducted with a dual reporter assay system. Briefly, the NF-κB vector was co-transfected with reference *Renilla *luciferase vector (Promega, Madison, WI, USA) ast a ratio of 10:1. After transfection for 48 h, cells were lysed by passive lysis buffer (Promega) and luciferase activity was detected with Beetle-Juice (for firefly luciferase (FLuc)) and Gaussia-Juice (for Renilla luciferase (RLuc)) substrates (PJK GmbH, Kleinblittersdorf, Germany) and luminescence was counted with luminescence reader (Promega). The results of FLuc count were normalized with RLuc, which represented the transfection efficiency of each sample.

## Results

### Up-regulation of Hsp27 and its phosphorylation in breast cancer stem cells

**Figure 1:**
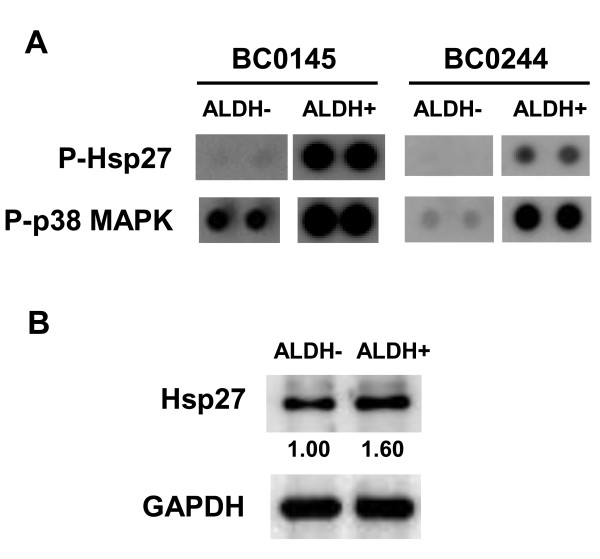
**Increased expression of Hsp27 in ALDH+ BCSCs**. (**A**) ALDH- or ALDH+ cells were sorted from BC0145 and BC0244 xenografts and 150 μg of extracted total protein were subjected into PVDF membrane of MAPK array as described in "Materials and methods" section. P-Hsp27, phosphorylated Hsp27; p-p38 MAPK, phosphorylated p38 MAPK. (**B**) ALDH- or ALDH+ cells were sorted from AS-B244 cells and 25 μg of total protein were used for Hsp27 or GAPDH detection by Western blot. Inserted values indicated relative expression of Hsp27 in ALDH- or ALDH+ cells. ALDH, aldehyde dehydrogenase; MAPK, mitogen activated protein kinase.

We have previously established two human breast cancer cells from xenografts of NOD/SCID mice and identified that cells with high intracellular aldehyde dehydrogenase activity (ALDH+) are cancer stem cells [14]. With ALDH activity, proteins extracted from ALDH- or ALDH+ cells of BC0145 or BC0244 xenografts were subjected to mitogen activated protein kinase (MAPK) antibody array and results indicated that the activation of Akt, ERK, p38 MAPK and RSK1 were increased in BSCSs (Additional file 1 Figures S1 and S2). The phosphorylation of Hsp27, which may result from p38 MAPK activity, was also increased in ALDH+ BCSCs from BC0145 or BC0244 xenograft cells (Figure 1A). We also used Western blot to check the level of total Hsp27 protein between ALDH- and ALDH+ AS-B244 cells, which derived from ALDH+ BC0244 xenograft cells [14]. As shown in Figure 1B, the total protein level of Hsp27 was higher in ALDH+ cells than in ALDH- cells. These results indicate that Hsp27and its phosphorylation are up-regulated in BCSCs.

### Hsp27 determines the maintenance of breast cancer stem cells as well as their characteristics of epithelial-mesenchymal transition

**Figure 2:**
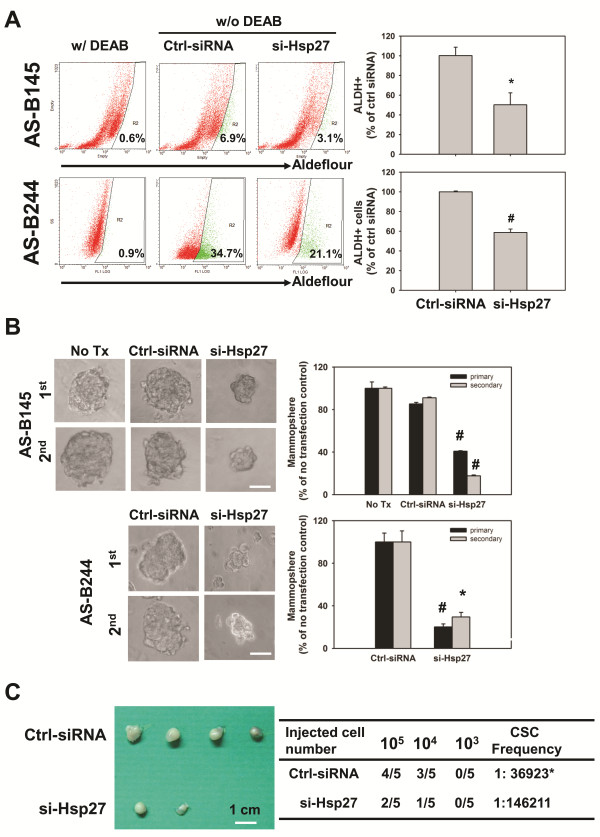
**Knockdown of Hsp27 decreased BCSCs of breast cancer cells**. (**A**) AS-B145 cells were transfected with Hsp27 siRNA (si-Hsp27) or negative control siRNA (ctrl-siRNA) for 48 h and detected a ALDH+ population by ALDEFLUOR assay. The percentage of ALDH+ cells were calculated from samples without DEAB treatment (w/o DEAB) after gating with a cutoff line which was set according to DEAB treated (w/DEAB) cells. The quantitative results were presented as relative percentage of ctrl siRNA transfected cells. *, *P *< 0.05. (**B**) Primary (1^st^) mammosphere formation capacity of AS-B145 or AS-B244 cells was also analyzed after knockdown of Hsp27 for 48 h. For secondary (2^nd^) mammosphere cultivation, 1^st ^spheres were collected, digested with accutase and subjected to mammosphere cultivation at 48 h post-transfected with si-Hsp27 or negative control siRNA (ctrl-siRNA). Data were collected at Day 7 post starting the cultivation and presented as relative percentage of ctrl siRNA. Bar, 100 μm. #, *P *< .01; *, *P *< 0.05. (**C**) The tumorigenicity of AS-B145 sphere cells after knockdown of Hsp27 (si-Hsp27) or negative control siRNA (ctrl-siRNA) was determined by xenograftment assay in NOD/SCID mice. The indicated numbers of cells were injected into mammary fat pads for 44 days and the tumor formation was monitored weekly. The cancer stem cell (CSC) frequency was calculated with ELDA software [15]. *, *P *< 0.05. ALDH, aldehyde dehydrogenase; BCSCs, breast cancer stem cells.

**Figure 3:**
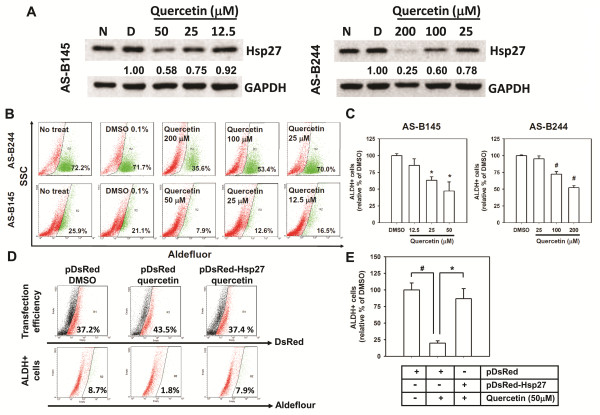
**Quercetin dose-dependently decreased Hsp27 expression and ALDH+ population of breast cancer cells**. (**A**) AS-B145 or AS-B244 cells were treated with quercetin at indicated concentration for 48 h and detected the expression of Hsp27 by Western blot. Inserted values indicated relative expression of Hsp27 in comparison with DMSO. N, no treatment; D, DMSO. (**B, C**) ALDH+ population after treatment of quercetin was determined by ALDEFLUOR assay. The quantitative results were shown in bar graph (C). #, *P *< 0.01; *, *P *< 0.05. (**D, E**) AS-B145 cells were transfected with pDsRed-Express2-C1 (pDsRed) or pDsRed-Hsp27 and treated with 0.1% DMSO or 50 μM quercetin for 48 h. The transfection efficiency of each group was approximately 40% (upper panel) and red fluorescence positive cells were gated to further analyze Aldefluor green fluorescence signal (lower panel) by WinMDI software. The quantitative results were shown in bar graph (**E**). *P *< 0.01; *, *P *< 0.05. ALDH: aldehyde dehydrogenase.

**Figure 4:**
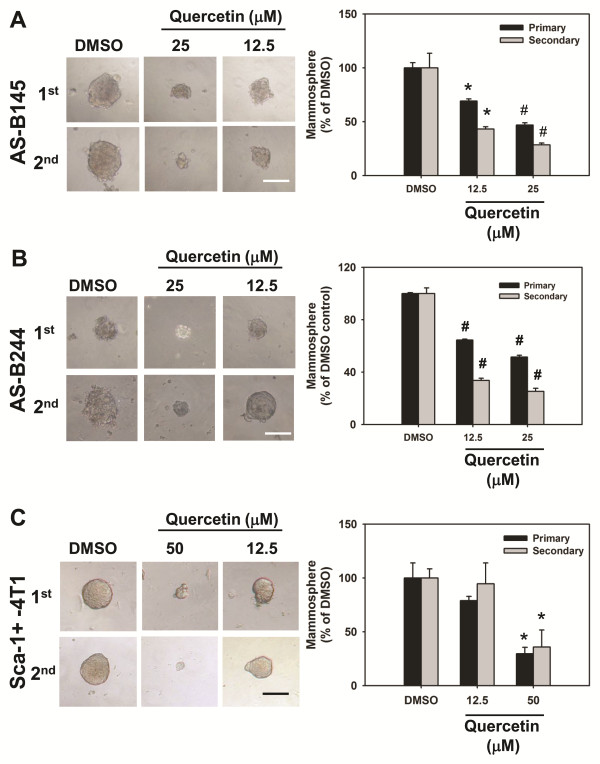
**Quercetin dose-dependently inhibited self-renewal of human and mouse breast cancer cells**. 2 × 10^4 ^cells/ml of AS-B145 (**A**), AS-B244 cells (**B**) or Sca-1+ 4T1(**C**) were subjected into primary (1^st^) mammosphere culture in present of quercetin (50, 25 or 12.5 μM) or 0.1% DMSO. For secondary mammosphere (2^nd^) cultivation, 1^st ^spheres were collected, digested by accutase and 5,000 cells/ml were subjected into mammosphere cultivation in present of quercetin or DMSO. Data were collected at Day 7 after the start of cultivation and presented as a relative percentage of DMSO. Bar in (A), 100 μm; bar in (B), 50 μm; bar in (C), 200 μm. *, *P *< 0.05; #, *P *< 0.01.

We next investigated the role of Hsp27 in maintenance of BCSCs by siRNA mediated gene silence of Hsp27 expression. After transfection with Hsp27 specific siRNA, the population of ALDH+ cells in AS-B145 or AS-B244 cells was significantly decreased to (50.2 ± 12.2)% or (58.7 ± 3.5)%, respectively, when compared with cells transfected with negative control siRNA (Figure 2A). Knockdown of Hsp27 not obviously caused cell death (less than 22% of all time points) and slowed the cell growth rate of AS-B145 cells (Additional file 1 Figure S3A, C), but caused obvious cell death (35% and 65% at 72 h and 96 h, respectively) and decreased cell number at 72 h and 96 h in AS-B244 cells (Additional file 1 Figure 3B, D). Other than the ALDH+ population of cells, the number of mammospheres as well as the size of formed spheres in AS-B145 or AS-B244 cells were also decreased (Figure 2B). We further examined if Hsp27 was involved in the tumorigenicity of BCSCs. AS-B145 sphere cells were collected for seven days after mammosphere culture, transfected with negative control siRNA or Hsp27 specific siRNA for 48 h and injected into mammary fat pads of female NOD/SCID mice in a serial dilution of injected cell number. As shown in Figure 2C, 10^5 ^negative control siRNA transfected AS-B145 sphere cells formed tumors in four out of five mice but 10^5 ^Hsp27 knockdown cells only formed tumors in two out of five mice at Day 44 (Figure 2C, left panel). The CSC frequency of Hsp27 knockdown AS-B145 sphere cells was significantly decreased when compared with negative control siRNA groups (1:30,680 versus 1:146,211, *P *= 0.0206). In addition to RNA interference, we also used quercetin, a plant flavonoid compound which has been reported to suppress the protein level of Hsp27 [16], to treat AS-B145 and AS-B244 cells. Quercetin inhibited the expression of Hsp27 protein (Figure 3A) as well as the population of ALDH+ cells (Figure 3B) in both AS-B145 and AS-B244 cells in a dose-dependent manner (Figure 3C). In order to confirm if the inhibition effect of quercetin is mediated by down-regulation of Hsp27, we next overexpressed Hsp27 in AS-B145 cells and examined the ALDH+ population under quercetin treatment. As shown in Figure 3D and 3E, the inhibitory effect of quercetin could be reversed by overexpression of Hsp27 in AS-B145 cells. We next tested if quercetin also inhibits the self-renewal of BCSCs by mammosphere formation assay. The size and number of primary and secondary mammospheres in AS-B145 (Figure 4A) and AS-B244 (Figure 4B) was suppressed by quercetin in a dose-dependent manner. In addition to human BCSCs, we also tested if quercetin could inhibit self-renewal of Sca-1+ 4T1 mouse BCSCs [17]. As shown in Figure 4C, quercetin decreased primary and secondary mammosphere formation of Sca-1+ 4T1 cells in a dose-dependent manner.

**Figure 5:**
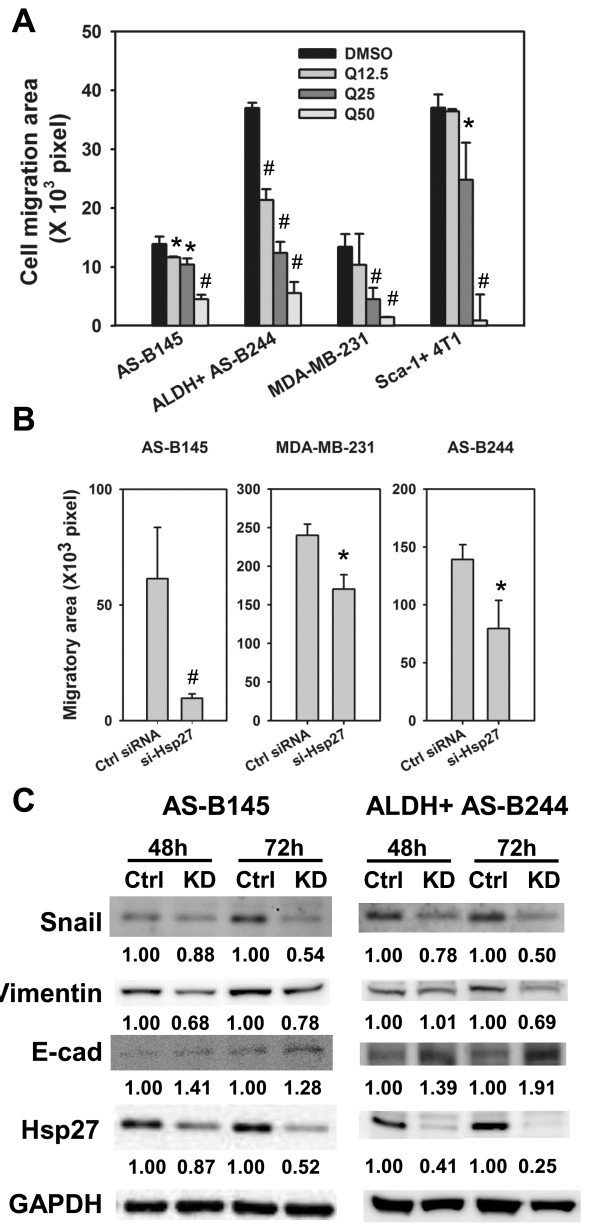
**Inhibition of Hsp27 suppressed EMT signature of BCSCs**. (**A**) Cell migration ability of AS-B145, ALDH+ AS-B244, MDA-MB-231 or Sca-1+ 4T1 cells in present of quercetin (Q, 12.5, 25 or 50 μM) or 0.1% DMSO or was analyzed with wound-healing assay. *, *P *< 0.05; #, *P *< 0.01. (**B**) Cell migration ability of AS-B145, MDA-MB-231 or ALDH+ AS-B244 cells after transfection of Hsp27 siRNA (si-Hsp27) or negative control siRNA (ctrl siRNA) was analyzed with wound-healing assay. *, *P *< 0.05; #, *P *< 0.01. (**C**) EMT related proteins (E-cadherin, vimentin or snail) of AS-B145 or ALDH+ AS-B244 cells were analyzed by Western blot. Inserted values indicated relative expression of proteins in negative control siRNA (ctrl) or si-Hsp27 (KD) transfected cells. ALDH: aldehyde dehydrogenase; BCSCs: breast cancer stem cells; EMT, epithelial-mesenchymal transition.

EMT is an important character of cancer stem cells [18]. We next examined if Hsp27 mediates EMT features of BCSCs. With a wound healing based cell migration assay, the cell migration ability of ALDH+ AS-B244, AS-B145, MDA-MB-231 and Sca-1+ 4T1 cells was inhibited by quercetin treatment in a dose-dependent manner (Figure 5A). Furthermore, quercetin treatment dose-dependently inhibited the expression of N-cadherin and twist but increased E-cadherin expression in both AS-B145 and ALDH+ AS-B244 cells (Additional file 1 Figure 4). By siRNA mediated knockdown of Hsp27, the cell migration capacity of AS-B145, MDA-MB-231 or ALDH+ AS-B244 cells was also inhibited in comparison with negative control siRNA (Figure 5B). We also investigated if the Hsp27 pathway also regulates EMT-related molecular signatures. With Western blot analysis, knockdown of Hsp27 in AS-B145 or ALDH+ AS-B244 cells decreased the expression of snail and vimentin and increased the expression of E-cadherin (Figure 5C). These results indicate that Hsp27 may regulate self-renewal of BCSCs through manipulating the EMT process.

### Hsp27 contributes to IκBα degradation and NF-κB activation in breast cancer stem cells

**Figure 6:**
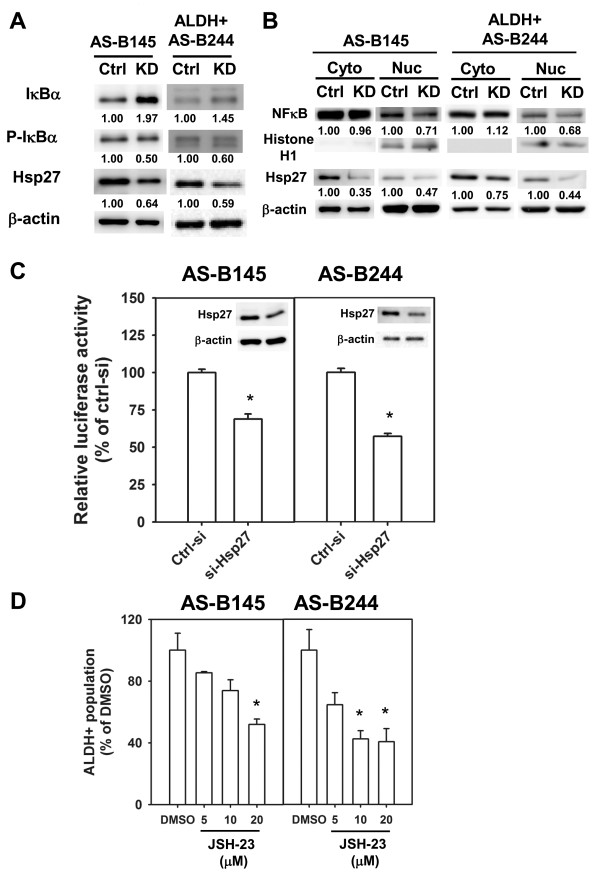
**Knockdown of Hsp27 decreased the activity of NF-κB in BCSCs**. (**A**) AS-B145 or ALDH+ AS-B244 cells were transfected with negative control siRNA (Ctrl) or Hsp27 siRNA (KD) for 24 h or 48 h and total proteins were harvested to detect the expression of IκBα, phosphor-IκBα (p-IκBα), Hsp27 and β-actin by Western blot. Inserted values indicated relative proteins expression in comparison with ctrl siRNA. (**B**) AS-B145 or ALDH+ AS-B244 cells were transfected with negative control siRNA (Ctrl) or Hsp27 siRNA (KD) for 48 h and nuclear (Nuc) and cytosolic (Cyto) proteins were isolated to detect NF-κB, histone H1, Hsp27 and β-actin by Western blot. Inserted values indicated relative proteins expression in comparison with ctrl siRNA. (**C**) AS-B145 or ALDH+ AS-B244 cells were co-transfected with NF-κB reporter vector, Renilla luciferase vector and negative control siRNA (ctrl-si)/or Hsp27 siRNA (si-Hsp27) for 48 h. Luciferase activity was determined by dual luciferase assay. Data were presented as relative luciferase activity of ctrl siRNA. *, *P *< 0.05. (**D**) AS-B145 or AS-B244 cells were treated with JSH-23 (20, 10 or 5 μM) or 0.1% DMSO for 48 h and ALDH+ population were determined by ALDEFLUOR assay. Data were presented as relative percentage of DMSO. *, *P *< 0.05; #, *P *< 0.01. ALDH, aldehyde dehydrogenase; BCSCs, breast cancer stem cells; NF-κB, nuclear factor kappa B

**Figure 7:**
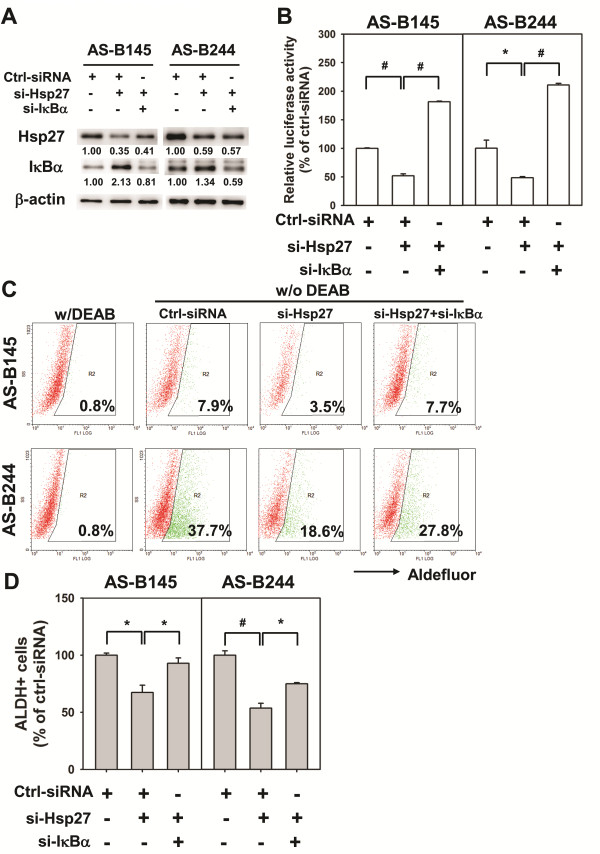
**Restoring NF-κB activity attenuates the suppressive effect of Hsp27 knockdown in ALDH+ breast cancer cells**. AS-B145 or AS-B244 cells were transfected with negative control siRNA (Ctrl-siRNA, 100 nM), ctrl-siRNA (50 nM)+si-Hsp27 (50 nM) or si-Hsp27 (50 nM) +si-IκBα (50 nM) for 48 h. (**A**) The expression of Hsp27 and IκBα was determined by Western blot. Inserted values indicated relative expression in comparison with ctrl-siRNA. (**B**) The NF-κB activity was determined by co-transfected with NF-κB-Luc reporter vector (0.5 μg) and RLuc (0.05 μg) vector. The results were presented as relative luciferase activity to ctrl-siRNA. (**C, D**) The percentage of ALDH+ population among groups was determined by ALDEFLUOR assay. DEAB was used to set the cutoff line for ALDH+ cells. The quantitative results were presented as relative percentage of negative control siRNA (ctrl-siRNA) group and were shown in bar graph (D). *, *P *< 0.05; #, *P *< 0.01. ALDH, aldehyde dehydrogenase; NF-κB, nuclear factor kappa B.

It has been reported that Hsp27 enhances the degradation of ubiquitinated proteins by 26S proteasome. Among these ubiquitinated proteins, phosphorylated IκBα could form a complex with Hsp27 and 26S proteasome and Hsp27 could enhance NF-κB activity by facilitating proteasome mediated IκBα degradation [19]. Recently, the NF-κB pathway has been demonstrated to participate in mammary tumorigenesis and cancer stem cell expansion in a transgenic mouse model [20]. We next examined if Hsp27 regulates NF-κB activity in BCSCs. By siRNA mediated knockdown of Hsp27, the expression of IκBα was increased in both AS-B145 and ALDH+ AS-B244 cells and its phosphorylation was decreased (Figure 6A). The nuclear translocation of NF-κB was also inhibited in both AS-B145 and ALDH+ AS-B244 cells when knockdown of Hsp27 occurred (Figure 6B). In the meantime, we also observed that Hsp27 could enter into the nucleus (Figure 6B). With a luciferase based reporter assay, the NF-κB activity was decreased in ALDH+ AS-B244 and AS-B145 cells when knockdown of Hsp27 occurred (Figure 6C). We next used NF-κB inhibitors to examine their effects on BCSCs. In the presence of JSH-23, a NF-κB inhibitor which inhibits the nuclear translocation of NF-κB, the ALDH+ population of AS-B145 and AS-B244 cells was suppressed in a dose-dependent manner (Figure 6D). We further examined if additional activation of NF-κB could diminish the inhibitory effect of ALDH+ cells by Hsp27 knockdown. The increased IκBα, which was caused by knockdown of Hsp27, was suppressed by knockdown of IκBα (Figure 7A) and the NF-κB activity could be restored in Hsp27 knockdown of AS-B145 or AS-B244 cells (Figure 7B). The inhibitory effect of ALDH+ cells by Hsp27 knockdown could be reversed by additional knockdown of IκBα in both AS-B145 and AS-B244 cells (Figure 7C, D). These results suggest that Hsp27 regulates the maintenance of BCSCs through NF-κB activity.

## Discussion

Hsp27 belongs to the small heat shock proteins and functions in an ATP-independent fashion. There are three phosphorylation sites of Hsp27, including serine 15, serine 78 and serine 84 [21]. The phosphorylation of Hsp27 leads the dissociation of the large Hsp27 chaperon complex into small Hsp27 dimer or tetramer and changes the chaperon activity into a cell signaling player [22]. The phosphorylation of Hsp27 has been demonstrated to contribute to many cellular behaviors of cancer, such as actin filament dynamics, cell survival, cell migration/invasion and cell differentiation [21]. For example, attenuation of Hsp27 phosphorylation by the specific microtubule inhibitor, KIRBB3, leads to a decrease in tumor cell migration and invasion [22]. In addition, Bausero *et al*. have demonstrated that the silencing of Hsp25 expression abrogated the migration potential of 4T1 cells through repression of matrix metalloproteinase 9 and up-regulation of tissue metalloproteinase 1 [23]. The phosphorylation of Hsp27 often affects its interaction with the target proteins. For example, the binding of tropomyosin with Hsp27 was increased when Hsp27 was phosphorylated [24]. In our study, Hsp27 phosphorylation in AS-B145 and AS-B244 was found at all three of these serine sites (unpublished observation). The role of Hsp27 phosphorylation in self-renewal or EMT character of BCSCs should be further investigated by overexpression of phosphor-mimic (serine change to aspartic acid) or phosphor-dead (serine change to alanine) mutants.

Hsps are widely known for their cytoprotection functions in cancer cells [25]. These mechanisms include their molecular chaperone activity, anti-apoptosis function and influence on the stability of client proteins [26]. Many Hsp27 client proteins have been reported previously [27]. For example, Hsp27 binds with cytochrome c to inhibit apoptosis [28]. In our study, knockdown of Hsp27 in breast cancer cells did not induce marked cell death at 48 h, which was the time point at which we analyzed the ALDH+ population in both AS-B145 and AS-B244 cells, but slowed the cell growth (Additional file 1 Figure S3). It suggests that the clients of Hsp27 in BCSCs possibly include proteins which are not related to apoptosis. It has been reported that Hsp27 enhances the degradation of ubiquitinated proteins, such as phosphor-IκBα, by 26S proteasome [19]. Here we also found that Hsp27 could regulate the nuclear translocation and activity of NF-κB in ALDH+ BCSCs through increasing the expression of IκBα (Figure 6). The 26S proteasome mediates protein degradation not only in IκBα, but also in p53, which is a suppressor of self-renewal of BCSCs [29]. Lagadec *et al*. have demonstrated that BCSCs could be defined as cells with low 26S proteasome activity [30]. Our observations suggest that Hsp27 might enhance the degradation of self-renewal suppressors in BCSCs, which are cells with low proteasome activity in their cellular microenvironment. It is well known that NF-κB is an important transcriptional factor in the expression of cytokines, including IL-6 and IL-8. High serum IL-6 concentration has been reported to be correlated with poor prognosis for breast cancer [31]. IL-6 mRNA expression has been shown to be evaluated in mammospheres derived from malignant mammary tissues [32]. Moreover, IL-6 autocrine loop could trigger a Notch-3/Jagged-1 pathway to enhance the growth and aggressive phenotypes of mammospheres derived from malignant mammary tissue or MCF7 breast cancer cell line [32]. On the other hand, IL-8/CXCR1 signal has been demonstrated to maintain the self-renewal of BCSCs [33]. IL-8 expression was increased in mammospheres and treatment of IL-8 increased the mammosphere number of breast cancer cells and blockage of CXCR1 signaling by repertaxin reduced chemoresistance of BCSCs [33]. Because Hsp27 regulates the activity of NF-κB in BCSCs, it is possible that Hsp27 is also involved in the regulatory function of IL-6 and IL-8 on BCSCs.

HSPs have also been found on cell membrane. Glucose-related protein (GRP)-78, a member of Hsp70 family, has been demonstrated as a novel marker of CSCs of head and neck squamous carcinoma cells (HNSCC) [34]. Knockdown of GRP78 reduced self-renewal ability and expression of stemness genes but induced differentiation and apoptosis of CSCs of HNSCC [34]. In mouse breast cancer 4T1 study, Hsp25 (an Hsp27 homolog in mouse) could be detected on the cell surface. With FACS, 4T1 cells with surface Hsp25+Hsp70- displayed high tumorigenicity and metastatic ability when compared with surface Hsp25+ Hsp70+ cells [35]. It suggests that surface Hsp25+ Hsp70- could serve as a marker of 4T1 CSCs. Whether Hsp27 could also be expressed on the cell surface of breast cancer cells and as a novel marker for BCSCs, should be investigated further.

## Conclusions

In conclusion, our present study demonstrates that Hsp27 participates in the maintenance of BCSCs, which were determined by mammosphere forming capability and cell migration potential of breast cancer cells and ALDH+ BCSCs. Hsp27 also is involved in the activation of NF-κB in breast cancer cells and ALDH+ BCSCs by regulation of IκBα degradation. These mechanisms demonstrate how Hsp27 contributes to the maintenance of BCSCs. Hsp27 inhibitors, such as quercetin, can potentially be developed in chemoprevention of breast cancer.

## Abbreviations

ALDH: aldehyde dehydrogenase; BCSCs: breast cancer stem cells; DEAB: diethylaminobenzaldehyde; EMT: epithelial-mesenchymal transition; ERK: extracellular regulated kinase; HSPs: heat shock proteins; MAPK: mitogen activated protein kinase; NF-κB: nuclear factor kappa B; RSK: ribosomal protein S6 kinase.

## Competing interests

The authors declare that they have no competing interests.

## Authors' contributions

WL, TTL, HHW, HHM and HPF conducted all the experiments. AY provided the breast cancer xenografts and cell lines, and helped with the experimental designs. WWC participated in the experimental designs, conducted all the experiments and drafted the manuscript. All the authors read and approved the manuscript for publication.

## Supplementary Material

Additional file 1 **Supplementary Figure S1, Figure S2, Figure S3 and Figure S4**. The additional file 1 contains (1) the raw data of mitogen-activated protein kinase antibody array of two xenograft tumor cells (Fig. S1 and Fig. S2); (2) growth curve and cell viability data of Hsp27 knockdown breast cancer cells (Fig. S3); and (3) western blot results of the expression of EMT related proteins in quercetin treated breast cancer cells or ALDH1+ BCSCs (Fig. S4). Click here for file
