# Supplementary material for: Hsp27 participates in the maintenance of breast cancer stem cells through regulation of epithelial-mesenchymal transition and nuclear factor-κB
Source: Breast Cancer Res. 2011 Oct 24;13(5):R101. doi: 10.1186/bcr3042 (PMC3262214; doi:10.1186/bcr3042)
Supplement: Additional file 1 — Supplementary Figure S1, Figure S2, Figure S3 and Figure S4. The additional file 1 contains (1) the raw data of mitogen-activated protein kinase antibody array of two xenograft tumor cells (Fig. S1 and Fig. S2); (2) growth curve and cell viability data of Hsp27 knockdown breast cancer cells (Fig. S3); and (3) western blot results of the expression of EMT related proteins in quercetin treated breast cancer cells or ALDH1+ BCSCs (Fig. S4). [file bcr3042-S1.DOC]

**Additional files**

**Figures**


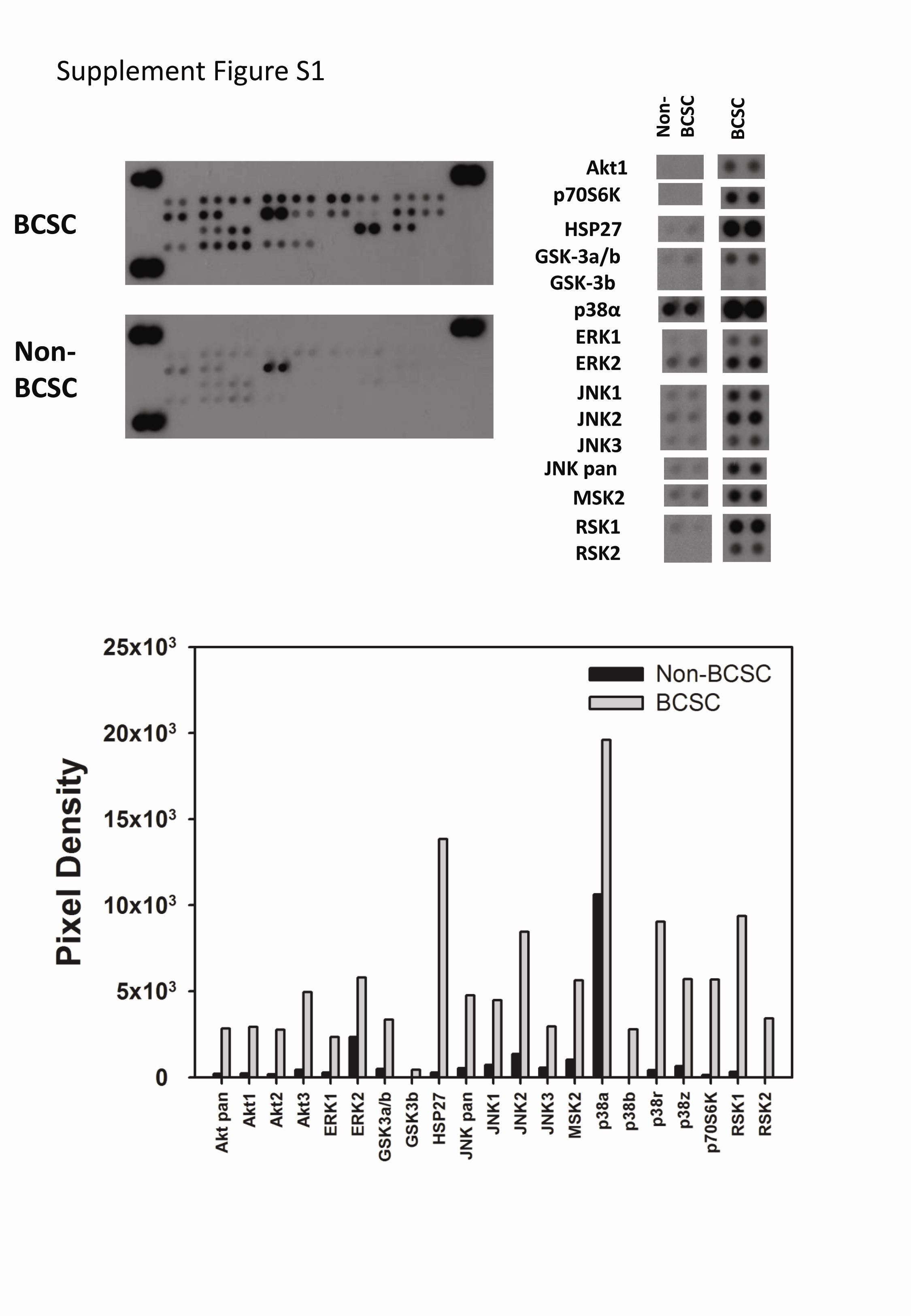


**Figure S1. MAPK array data of ALDH- or ALDH+ cells from BC0145 human breast cancer xenograft.** 150μg of total proteins of ALDH- (non-BCSCs) or ALDH+ (BCSCs) cells were subjected into MAPK array (R&D System) as described in “Materials and Methods” section. Intensities of dot signals were determined by ImageJ software.


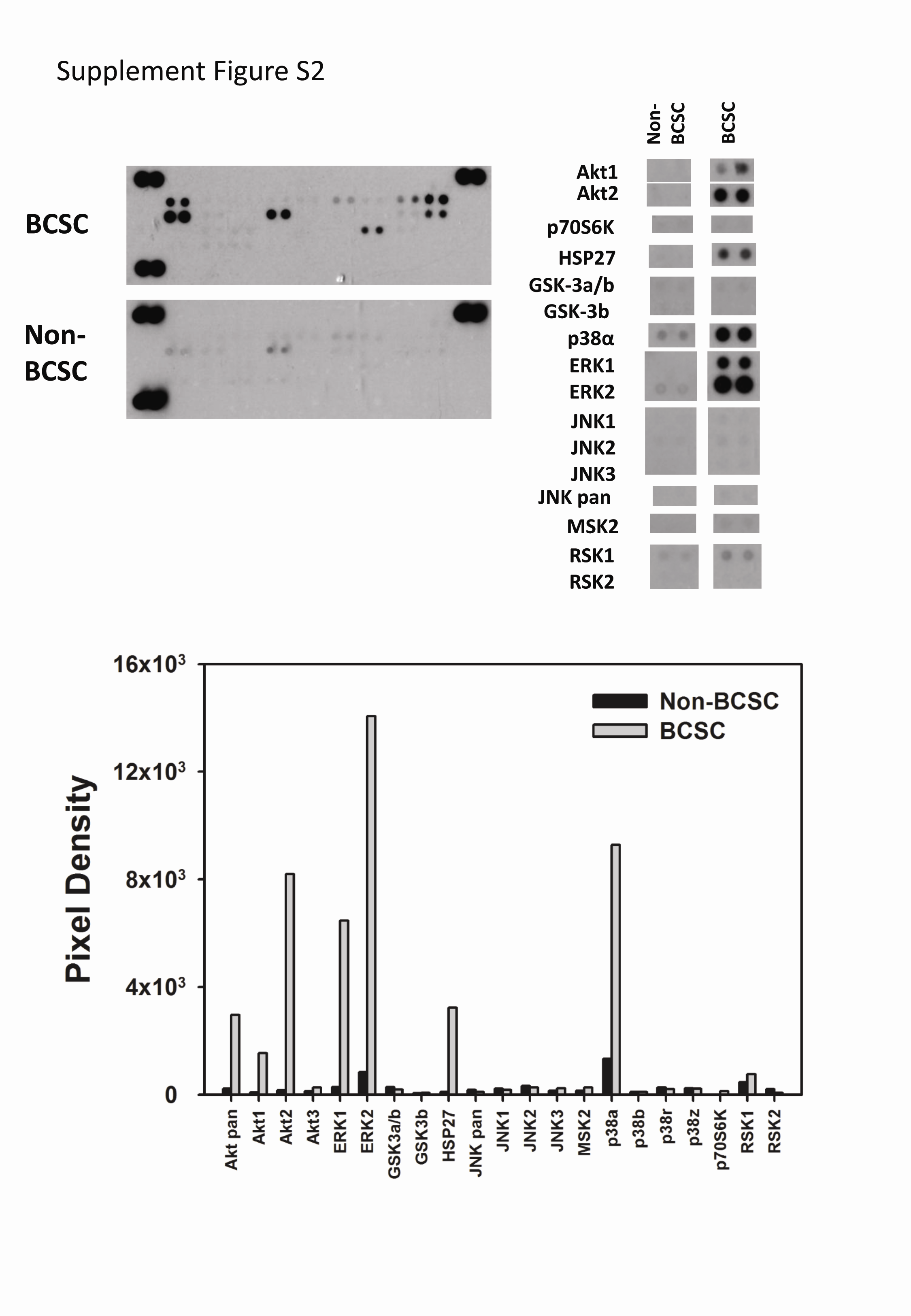


**Figure S2. MAPK array data of ALDH- or ALDH+ cells from BC0244 human breast cancer xenograft.** 150μg of total proteins of ALDH- (non-BCSCs) or ALDH+ (BCSCs) cells were subjected into MAPK array (R&D System) as described in “Materials and Methods” section. Intensities of dot signals were determined by ImageJ software.


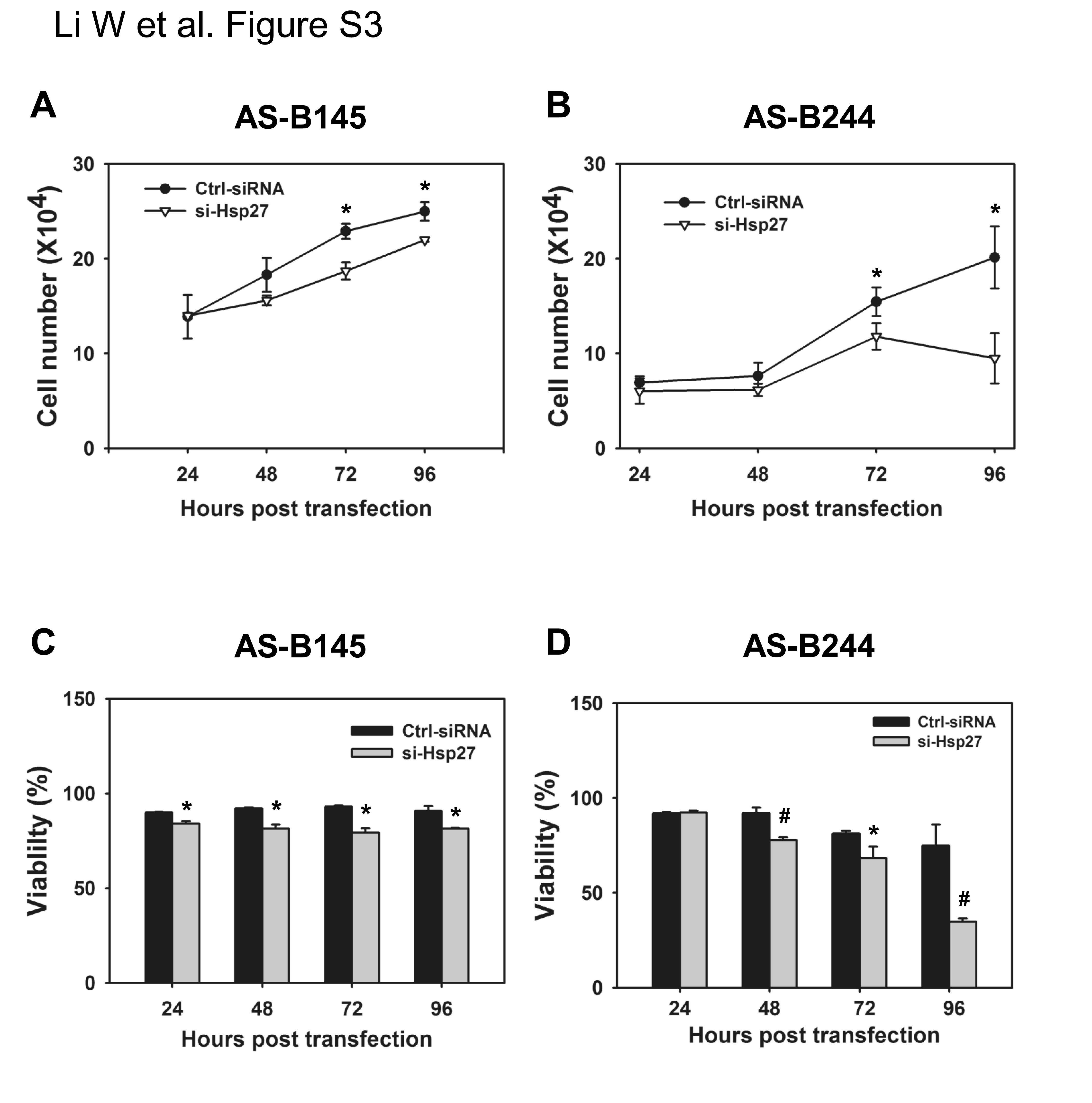


**Figure S3. Cell growth curve and viability of Hsp27 siRNA transfected AS-B145 cells and AS-B244 cells.** AS-B145 cells or AS-B244 cells were seeded as density of 1.0×10^5^ cells/well in 12-well-plate and transfected with negative control siRNA (ctrl- siRNA) or Hsp27 siRNA (si-Hsp27) at concentration of 100nM. Cells were harvested at indicated time points and cell number (A, AS-B145; B, AS-B244) and viability (C, AS-B145; D, AS-B244) were determined by trypan blue stain. *, p<0.05; #, p<0.01.


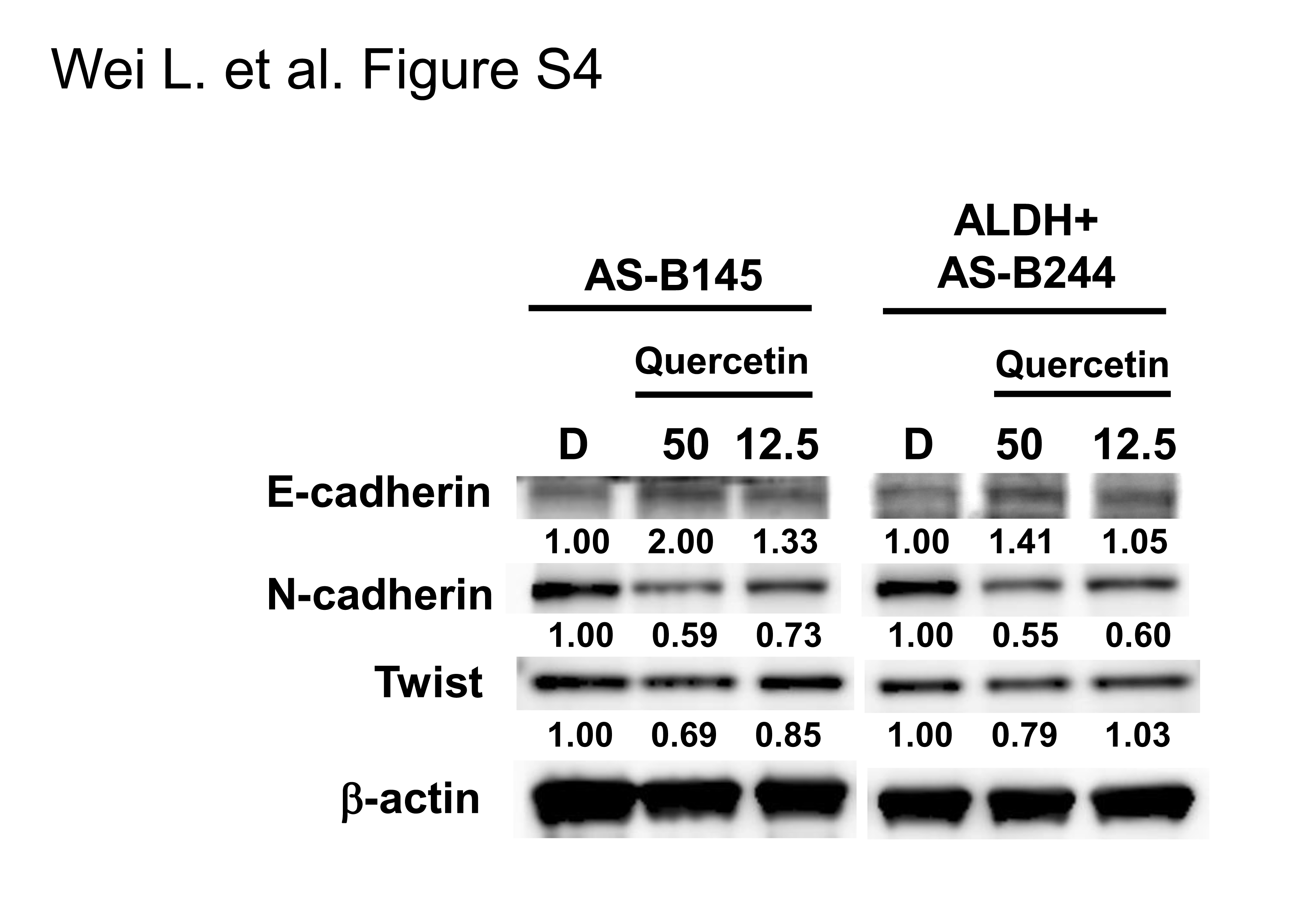


**Figure S4. Quercetin suppressed EMT signatures of AS-B145 or ALDH+ AS-B244 cells.** AS-B145 cells or ALDH+ AS-B244 cells were seeded as 2×10^5^ cells/well in 6-well-plate and treated with DMSO control or indicated concentration of quercetin (μM) for 24h. Cells were then harvested and lyszed with NP-40 lysis buffer and the EMT signatures (E-cadherin for epithelial marker, N-cadherin/twist for mesenchymal marker and β-actin for protein loading control) were analyzed by western blot. The band intensities of each group were first normalized with its own β-actin and then calculated the relative expression in comparison with DMSO control which were shown as the inserted numbers.
